# Cardiolipin and mitochondrial membrane integrity in neurodegeneration: insights from α-synuclein-driven Parkinson’s disease

**DOI:** 10.1186/s40478-025-02190-x

**Published:** 2025-12-03

**Authors:** Eva D. Ruiz-Ortega, Anna Wilkaniec, Josué Juárez, Agata Adamczyk

**Affiliations:** 1 Department of Cellular Signalling, Mossakowski Medical Research Institute, Polish Academy of Sciences, Warsaw, Poland; 2 Department of Physics, University of Sonora, 83000 Hermosillo, Mexico; 3 Department of Chemical Engineering and Metallurgy, University of Sonora, 83000 Hermosillo, Mexico

**Keywords:** Parkinson’s disease, Alpha-synuclein, Mitochondrial dysfunction, Cardiolipin, Liquid‒liquid phase separation

## Abstract

Parkinson’s disease (PD) is defined by the progressive loss of dopaminergic neurons and the accumulation of misfolded α-synuclein (α-syn), yet the molecular determinants of selective neuronal vulnerability remain unresolved. Increasing evidence implicates mitochondria—and particularly their membranes—as critical platforms where α-syn is toxic. This review highlights how α-syn engages mitochondrial membranes through two interconnected processes: classical aggregation and liquid‒liquid phase separation. Both pathways disrupt membrane architecture, compromise respiratory chain function, and impair mitophagy. A pivotal mediator of these events is cardiolipin (CL), a mitochondria-specific phospholipid essential for cristae organization and quality control pathways. Despite extensive progress, the precise mechanistic contributions of CL to α-syn aggregation, phase transitions, and neuronal degeneration remain poorly defined. Clarifying this interplay is crucial, as CL not only binds α-syn with high affinity but also determines whether it remains in a functional state or progresses toward toxic assemblies. By integrating recent advances, we propose a unifying perspective on CL as a molecular switch at the crossroads of mitochondrial biology, protein aggregation, and phase behavior. Beyond mechanistic insight, this view underscores the potential of CL as a target for the development of mitochondria-directed therapies in PD.

## Introduction

The pathological accumulation of misfolded proteins into insoluble, amyloid-like fibrils is a unifying hallmark of several major neurodegenerative disorders, including Alzheimer’s disease (AD), Parkinson’s disease (PD), multiple system atrophy (MSA), and amyotrophic lateral sclerosis (ALS). Each disorder arises from the misfolding and aggregation of distinct proteins within the central nervous system (CNS), resulting in distinctive molecular signatures and neuropathological features that enable their classification [39, 82, 223, 282]. Among these conditions, PD stands out as the second most common neurodegenerative disorder worldwide [51, 125, 211], and a major health challenge in aging populations [150], with a prevalence affecting 1–2% of individuals over the age of 65 [184]. The global burden of PD exceeded 11 million individuals in 2021, and demographic projections forecast a further 1.5-fold increase by 2035 [150], underscoring the urgent need for effective disease-modifying strategies.

PD is classified as an α-synucleinopathy [39, 56, 83, 120, 132, 158, 187, 193, 227, 249], a group of disorders characterized by the central accumulation and spread of α-synuclein (α-syn) in the form of Lewy pathology (LP), including Lewy bodies (LBs) and Lewy neurites (LNs) [61, 83, 120, 193, 227, 249]. Clinically, PD manifests as the most prevalent movement disorder [51, 211] and is driven by the degeneration of dopaminergic neurons in the substantia nigra pars compacta (SNpc). This neuronal loss underlies the cardinal motor symptoms—bradykinesia, tremor, rigidity, and postural instability [10, 55, 61, 90, 158, 184, 211, 215, 224]—which are often preceded by a long prodromal phase marked by nonmotor symptoms [111, 205]. Despite this heterogeneity of presentation, the definitive diagnosis still relies on the postmortem identification of LP [85].

Mounting evidence underscores the pathogenic role of aggregated α-syn and its intercellular propagation in PD [164]. Furthermore, mitochondria have emerged as a central hub in this process, providing a platform where pathological α-syn converges with immune dysregulation, oxidative stress, and neuroinflammatory responses. Disruptions in mitochondrial dynamics—fusion, fission, and selective removal of damaged organelles—are increasingly recognized as key drivers of neuronal vulnerability [31, 36, 37, 81, 123, 236, 274, 275]. Within this context, a functional relationship has been proposed between α-syn, lipid metabolism, membrane lipid composition [4, 13, 58, 64, 207, 212, 287], liquid–liquid phase separation (LLPS) [71, 200, 281], and mitochondrial function in the progression of neurodegeneration [11, 77, 79, 135, 147, 201, 212, 246, 268]. Together, these interconnected processes suggest that mitochondria are not passive targets of α-syn toxicity but active players that may shape protein behavior, determining whether misfolded α-syn remains confined, transitions into condensates, or matures into fibrillar inclusions.

Perturbations in lipid metabolism appear to predispose cells to α-syn accumulation and aggregation by altering lipid composition and amplifying the oxidative damage caused by lipid peroxidation [246]. For example, increased levels of polyunsaturated fatty acids (PUFAs), such as arachidonic acid (AA) [105], docosahexaenoic acid (DHA) [62, 63], and cholesterol [156], have been linked to increased α-syn aggregation [203]. Moreover, interactions between α-syn and oxidized lipid metabolites exacerbate mitochondrial damage and organelle dysfunction, thereby creating a feed-forward loop that promotes pathology [202].

Among mitochondrial lipids, cardiolipin (CL, 1,3-bis(sn-3′-phosphatidyl)-sn-glycerol) occupies a particularly intriguing position. Exclusively localized to the mitochondria, CL maintains the integrity of the mitochondrial membrane and orchestrates key processes, including respiration, crista organization, and mitophagy. Its dysregulation has been proposed to compromise membrane permeability, disrupt respiratory efficiency, and accelerate protein aggregation, ultimately driving neuronal loss [57, 239]. In addition to its structural roles, CL has recently been implicated in regulating LLPS within the mitochondrial environment, influencing the condensation and aggregation dynamics of α-syn [144, 186]. This emerging perspective places CL as a molecular switch at the crossroads of mitochondrial biology and protein misfolding. However, the precise mechanisms of CL-driven regulation remain largely unexplored, and clarifying them could open new avenues for therapeutic intervention in PD and related α-synucleinopathies [57].

## Conformational plasticity of α-syn

**Fig. 1:**
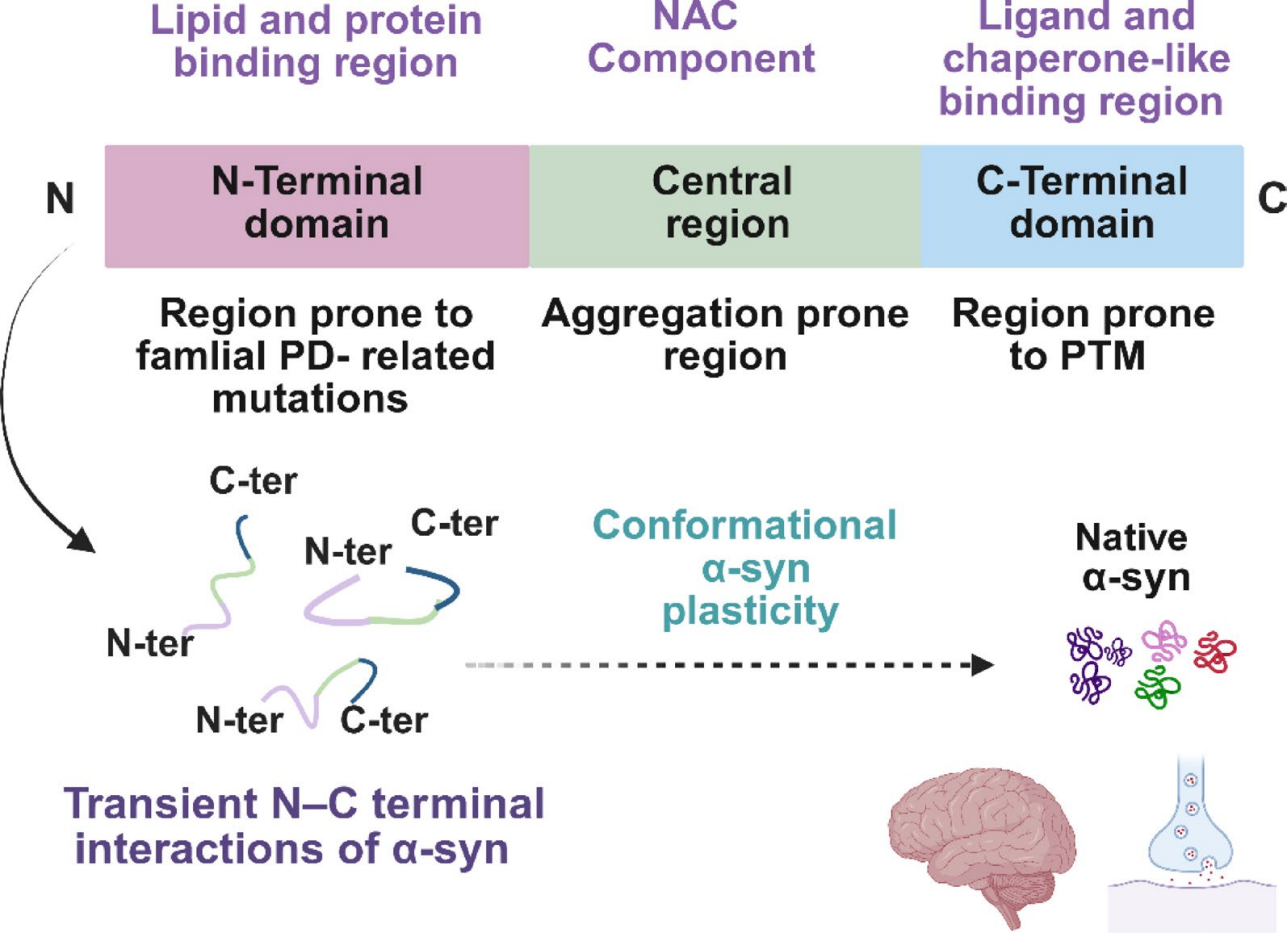
Conformational plasticity of monomeric α-syn. The primary structure of α-syn is divided into three domains: an N-terminal domain (pink), which is susceptible to familial mutations associated with PD; a highly hydrophobic central region, the nonamyloid-β component (NAC) (green), which drives protein aggregation; and a highly charged C-terminal domain (blue), a site of numerous posttranslational modifications. The conformational plasticity of α-syn, which is regulated by intramolecular interactions between the N- and C-terminal domains, is critical for its physiological functions, including the regulation of vesicular trafficking and neurotransmitter exocytosis. Created with BioRender.com

α-Syn is a 14 kDa protein whose primary structure is divided into three domains: the amphipathic N-terminal domain (residues 1–60), the central nonamyloid-β component (NAC, residues 61–95), and the highly charged C-terminal domain (residues 96–140) [243]. In aqueous solution, α-syn displays pronounced flexibility and lacks a stable three-dimensional structure [160, 288] (Fig. 1).

This intrinsically disordered structure allows the protein to continuously experiment with a wide range of conformations [134, 160], including α-helical, random coil, and partially folded states [134, 160, 233–235]. Electrostatic interactions between the positively charged N-terminal and negatively charged C-terminal domains, together with hydrophobic forces stabilizing the NAC region, are critical in shielding aggregation-prone β-sheet motifs [2, 118, 233, 235]. The N-terminus contains a lipid-binding α-helical motif [117, 132, 237], whereas the acidic C-terminal tail imparts solubility and prevents self-assembly and aggregation [257].

In addition to its canonical role in regulating synaptic vesicle trafficking and neurotransmitter release [101, 128], α-syn is increasingly recognized as a multifunctional protein involved in synaptic plasticity, mitochondrial dynamics, and neuronal survival [23]. To perform such diverse tasks, its conformational plasticity is essential. Interactions with highly curved, lipid-rich structures—most prominently synaptic vesicles and mitochondrial membranes—induce α-helical folding, thereby enabling transient and reversible membrane association [12, 22, 54, 73, 180]. This feature allows α-syn to act as a molecular sensor of membrane curvature and composition. However, this same plasticity creates vulnerability.

Under specific stress conditions—including high local concentrations, altered pH, oxidative stress, or exposure to metal ions—α-syn can shift from its disordered or helical conformations to β-sheet-rich assemblies, initiating fibrillization [1, 86, 280]. The ability of α-syn to toggle between states may help explain the heterogeneity of α-synucleinopathies: different conformational “strains” could preferentially engage with specific lipid environments, resulting in distinct pathological signatures across PD, MSA, and DLB.

A particularly intriguing, and still underexplored, aspect is the conformational crosstalk between α-syn and the mitochondrial CL. The N-terminal lipid-binding motif shows strong affinity for CL-rich domains, suggesting that the local mitochondrial lipid composition may dictate whether α-syn remains in a functional helical form or transitions toward toxic β-sheet aggregates. Thus, α-syn emerges not as an intrinsically pathogenic molecule but as a context-dependent protein whose structural fate is actively shaped by the surrounding lipid environment. This property provides the molecular foundation for its aggregation pathways.

## α-Syn aggregation: from classical pathways to LLPS and lipid-mediated mechanisms

**Fig. 2:**
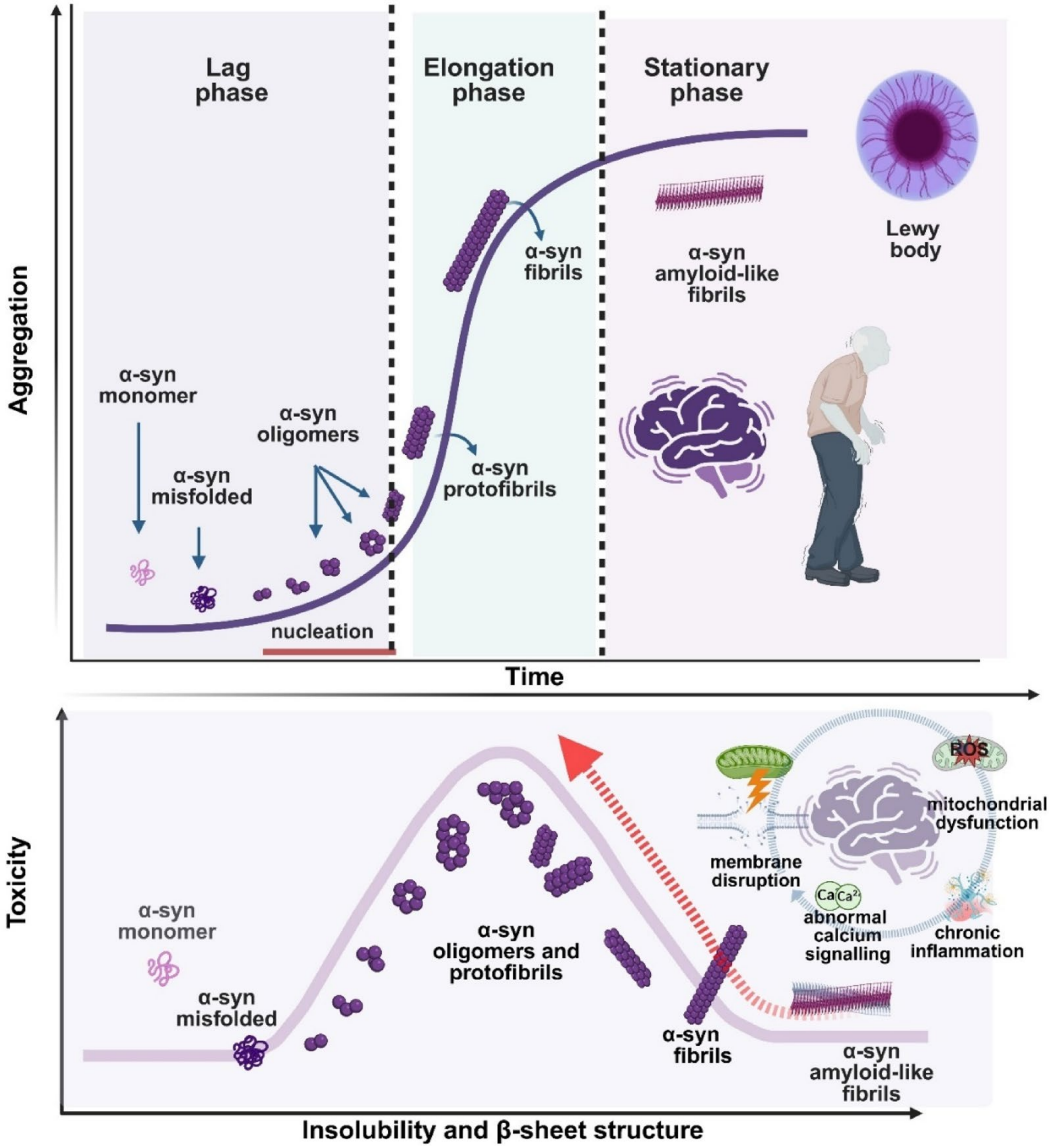
Model of the α-syn aggregation pathway and toxicity. The aggregation pathway (upper panel) comprises three distinct phases. In the lag phase, native monomeric α-syn undergoes conformational changes, misfolds, and forms aggregation-competent species (nuclei). This phase is thermodynamically unfavorable, and polymerization proceeds slowly. During the elongation phase, α-syn nuclei rapidly convert into oligomers, protofibrils, and fibrils. In the stationary phase, most soluble α-syn is transformed into insoluble amyloid-like fibrils that reach saturation. α-Syn toxicity is associated with changes in both α-syn solubility and β-sheet content (lower panel). Oligomers and protofibrils contribute primarily to toxicity by inducing cellular dysfunction—including mitochondrial impairment and chronic neuroinflammation—and cell death. α-Syn fibrils are prone to fragmentation, serving as a reservoir of toxic soluble oligomers (indicated by the red arrow) and generating new nucleation seeds that propagate the pathology. Created with BioRender.com

Experimental modelling of α-syn aggregation in vitro has provided a powerful framework for dissecting the molecular underpinnings of this process, yet many key aspects remain unresolved [213]. The use of recombinant preformed fibrils (PFFs) [197, 210, 218] or fibrils extracted directly from patients’ brain tissue [80, 130, 177, 213] has enabled the characterization of distinct aggregation pathways and the cytotoxic potential of different conformational states. Within this framework, α-syn aggregation has long been described by the classical nucleation–polymerization model [279]. In this case, soluble monomers undergo a conformational conversion to aggregation-competent nuclei (lag phase) [200, 223], followed by rapid polymerization into oligomers, protofibrils, and mature fibrils (elongation phase), culminating in a stationary phase where amyloid fibrils predominate [82, 83, 200, 223] (Fig. 2).

While this model explains the general kinetics of fibrillization, it fails to capture the complexity of α-syn species observed in human neuropathology. Indeed, oligomeric and protofibrillar assemblies, rather than mature fibrils, are widely accepted as the most neurotoxic α-syn assemblies [29, 34, 38–40, 55, 72, 145, 147, 255, 259, 273–275]. Structural variability among oligomers is substantial and plays a critical role in determining their toxic potential, as different conformational states can exert distinct effects on cellular homeostasis [51, 88, 158, 200, 223]. Certain oligomers, particularly those adopting cylindrical or annular shapes, exhibit strong interactions with lipid bilayers due to the exposure of hydrophobic β-sheet regions capable of inserting into membranes [70]. This insertion often results in pore-like disruptions that compromise membrane integrity and lead to ionic imbalance and calcium dysregulation [28, 158]. Other oligomers destabilize mitochondrial membranes and interfere with respiratory chain activity [27, 28, 49]. Consistently, cell toxicity has been correlated with the presence of β-sheet-rich structures, particularly those in an antiparallel arrangement by favouring membrane destabilization [28]. This suggests that oligomers displaying greater conformational flexibility and β-sheet exposure exhibit enhanced membrane binding and neurotoxicity. In contrast, larger fibrillar aggregates may act as sinks that sequester toxic species, yet their propensity to fragment provides new nucleation seeds that propagate pathology across neuronal networks [15, 72, 148, 149, 155, 264, 265]. This duality complicates therapeutic strategies, as interventions targeting fibrils may inadvertently liberate smaller, more toxic assemblies.

Further complexity arises from disease-specific conformations: multiple studies have demonstrated distinct α-syn structural strains in the brains of PD, MSA, and DLB patients [176, 278], suggesting that conformational diversity underlies clinical heterogeneity [177]. Lipids act as decisive modulators in this process. For example, interactions with phospholipids such as 2‐dimyristoyl‐sn‐glycero‐3‐phospho‐L‐serine (DMPS) [219], 1-palmitoyl-2-oleoyl-sn-glycero-3-phosphocholine (POPC), and 1-palmitoyl-2-oleoyl-sn-glycero-3-phosphate (POPA) [67] can remodel the α-syn ultrastructure and alter its cytotoxic potential [67, 219]. This evidence reinforces the notion that the lipid environment is not a passive backdrop but rather an active determinant of aggregation pathways.

Recently, attention has shifted toward an alternative aggregation mechanism—LLPS—which provides an additional layer of explanation [17, 42, 89, 115, 191, 228, 270, 294]. LLPS represents a physiological process in which proteins and nucleic acids condense into dynamic, membraneless compartments [3, 16, 84, 100, 267]. Aberrant LLPS and condensate formation have been associated with several human diseases, including neurodegenerative disorders such as AD [17, 89, 228, 270], PD [88, 100, 165, 191, 294], and ALS and frontotemporal dementia (FTD) [25]. Converging evidence indicates that similar LLPS-driven mechanisms also contribute to the pathogenesis of cancer [46, 103, 109, 142, 247, 267, 291], metabolic diseases [46, 267], and viral infections, including severe acute respiratory syndrome coronavirus 2 (SARS-CoV-2) [26, 96, 267]. In several of these conditions, lipids emerge as critical regulators of LLPS, providing membrane surfaces and localized chemical microenvironments that modulate condensate nucleation, maturation, and stability. Such lipid–condensate interactions can profoundly affect disease-relevant signaling pathways [46, 103, 108, 109, 142]. For example, cancer cells frequently enhance lipid droplet biogenesis and remodel lipid composition [108], thereby generating microdomains that facilitate the assembly of oncogenic condensates (e.g., metabolic enzyme clusters) or stabilize pro-survival mRNAs within stress granules. These processes collectively support malignant proliferation, treatment resistance, and metastatic potential [46, 108]. Furthermore, pathological crosstalk between aberrant condensates and mitochondrial dysfunction represents an additional determinant of tumor growth and survival [103, 142].

In the context of α-synucleinopathies, α-syn droplet formation via LLPS can precede fibrillization: initially dynamic condensates undergo a liquid-to-solid transition that promotes the emergence of oligomers and filamentous aggregates [88, 165, 191, 200, 294]. Importantly, LLPS may generate a spectrum of conformations with distinct toxicities, potentially explaining the clinicopathological variability of α-synucleinopathies [89, 102, 138, 183, 191]. In addition to the influence of lipids, protein quality control systems play a critical role in regulating the fate of these condensates. Molecular chaperones, particularly the heat shock protein Hsp70 together with DNAJ cochaperones (DNAJB1 and DNAJB6) and Hip (ST13), can suppress α-syn phase transitions and maintain condensates in fluid in a reversible state [136, 198, 277]. DNAJ proteins stimulate the adenosine triphosphatase (ATPase) activity of Hsp70, increasing substrate binding and promoting efficient suppression of α-syn aggregation. This cooperation is essential not only for preventing aggregation but also for actively disassembling fibrils through ATP-dependent “entropic pulling” [9, 163, 209, 271]. Loss of this surveillance markedly accelerates aggregation and toxicity, whereas posttranslational modifications (PTMs) of α-syn, such as phosphorylation at Tyr39, weaken chaperone binding and tilt condensates toward irreversible solidification [9, 21, 59]. Together, these findings emphasize that the outcome of LLPS is determined not only by physicochemical conditions but also by the efficiency of cellular chaperone networks, which act in parallel with lipid environments to define whether α-syn condensates remain functional or evolve into pathogenic assemblies. The importance of LLPS in PD development remains mostly unexplored, but its importance is substantial. Unlike the straightforward nucleation–polymerization process, LLPS provides a mechanism for α-syn to exist in metastable condensates, thereby balancing normal function and disease. This view suggests that mitochondrial membranes enriched in CLs may act as catalytic surfaces for condensate formation. By concentrating α-syn and altering its conformational equilibrium, CL-rich domains could determine whether LLPS remains reversible and physiological or progresses to irreversible aggregation. This perspective highlights a critical gap in our understanding: the interplay between lipid composition, condensate dynamics, and pathogenic aggregation. Defining how CL-rich membranes influence the balance between reversible condensates and irreversible fibrils may prove critical for explaining selective neuronal vulnerability in PD. Moreover, these findings could open new therapeutic avenues aimed at stabilizing physiological condensates or preventing their pathological conversion, thereby shifting the focus from downstream toxicity to the very origin of α-syn aggregation.

## The role of lipid membranes in α-syn pathology: the impact of mitochondrial membranes

Alterations in protein processing and cellular homeostasis profoundly affect the conformational behavior of α-syn and promote its aggregation into neurotoxic assemblies [24, 63, 66, 73, 94, 98, 112, 122, 139, 290]. PTMs, alternative splicing, mutations in the SNCA gene, and aberrant localization can all bias α-syn toward pathogenic states [24, 160]. Likewise, extrinsic factors such as pH, metal ions, polyamines [98, 122], proteoglycans [128], nucleic acids [94], and lipids [62, 63] may modulate folding and assembly, α-syn misfolding, and subsequent aggregation [281]. Among these modulators, lipids—particularly mitochondrial membranes enriched in CL—have attracted increasing attention as potential drivers of α-syn pathology in PD. The unique biochemical properties of these compounds suggest that understanding α-syn–lipid interactions could shed light on key mechanisms of aggregation and reveal novel therapeutic strategies.

## α-Syn–lipid interactions: linking membrane biology to mitochondrial dysfunction

The insoluble LP inclusions, the defining histopathological feature of PD, contain not only insoluble α-syn fibrils but also fragmented mitochondria, lysosomes, disrupted vesicles, and membranous debris [38, 58, 133, 206, 212]. This composition emphasizes that α-syn misfolding occurs within a lipid-rich, organelle-associated environment rather than in isolation. Indeed, mitochondrial dysfunction is a consistent correlate of α-syn pathology, although the underlying mechanisms remain incompletely resolved [87, 259]. On the one hand, aggregated α-syn perturbs protein homeostasis [157] and mitochondrial quality control [76], downregulating parkin and thereby promoting mitochondrial damage and oxidative stress [76, 274, 275]. On the other hand, α-syn interacts directly with mitochondrial membranes and associated proteins [114, 153, 188, 258, 259, 269], where oligomers form pore-like structures in lipid bilayers resembling the inner mitochondrial membrane (IMM), altering permeability and bioenergetics [251].

Lipids themselves are increasingly recognized as active participants in these processes. α-Syn not only binds membranes but also remodels them, altering both bilayer organization and vesicle stability [15, 49]. Reciprocally, lipids modulate α-syn structure and aggregation dynamics [29, 70, 79, 154, 161, 174, 246, 251, 254, 255]. Several mechanistic layers can be distinguished. Electrostatic interactions between the positively charged N-terminal region of α-syn and negatively charged headgroups, including phosphatidylserine and CL, initiate binding [43, 69]. Hydrophobic interactions involving the NAC region promote deeper insertion into bilayers, stabilizing α-helical conformations on curved membranes [70, 73]. α-Syn also senses and induces curvature, a property that facilitates oligomerization on vesicle surfaces [29, 161, 174, 251, 254, 255, 292]. Certain lipid species actively accelerate aggregation: anionic phospholipids such as CL and phosphatidylserine promote nucleation and fibril elongation [44, 70, 260, 263]. Conversely, oligomeric α-syn can compromise bilayer integrity by creating ion-permeable pores and driving calcium dysregulation [6, 24, 40, 129, 199, 262, 275].

Ultimately, lipid-induced conformational shifts bias α-syn toward either α-helical states or β-sheet assemblies [29, 43, 50, 70, 154, 161, 174, 251, 254, 255, 292]. Subtle modifications in mitochondrial lipids, including the fatty acid chain length, degree of saturation, and oxidative status, further tune these interactions [68, 73, 175, 214, 248]. Oligomers display high affinity for loosely packed, peroxidized membranes, where lipid oxidation accelerates their accumulation [79]. Electrostatic complementarity between charged headgroups and α-syn termini, combined with NAC-mediated hydrophobic insertion, reinforces these pathogenic assemblies [50]. This lipid remodelling not only enhances α-syn misfolding but also compromises mitochondrial resilience, resulting in a vicious cycle of oxidative stress and dysfunction.

Consistent with this view, spectroscopic analyses have demonstrated the incorporation of lipids into α-syn aggregates ex vivo [50], and experimental systems have shown that vesicles mimicking mitochondrial membranes accelerate oligomer formation and modulate their toxicity [43, 127]. Notably, α-syn aggregates formed in the presence of phosphatidylcholine were more cytotoxic to dopaminergic neurons than those formed under lipid-free conditions, highlighting the dual influence of lipid composition and secondary structure on toxicity [127]. Moreover, while zwitterionic lipids such as phosphatidylcholine can inhibit aggregation, anionic species such as phosphatidylserine dramatically accelerate aggregation [50, 127].

Collectively, these findings establish lipids as central modulators of α-syn pathology. They influence secondary structure, dictate aggregation kinetics, and shape the toxicity of resulting assemblies. Importantly, mitochondrial membranes enriched in CL appear to act as selective platforms where physiological binding transitions into pathological aggregation. Defining how these lipid–protein interactions govern the shift from functional membrane association to toxic assembly may therefore be essential for understanding selective neuronal vulnerability in PD and for developing strategies aimed at stabilizing protective conformations while preventing pathogenic conversion.

## Membrane platforms and CL in α-syn condensate formation and mitochondrial pathology

In recent years, increasing attention has been given to the interplay between mitochondria and α-syn, as α-syn contributes to mitochondrial homeostasis and dynamics under physiological conditions [60, 172, 188]. Even subtle perturbations of this interaction can trigger profound pathological consequences [45, 174], highlighting mitochondrial homeostasis as a promising therapeutic target in PD [285].

**Fig. 3:**
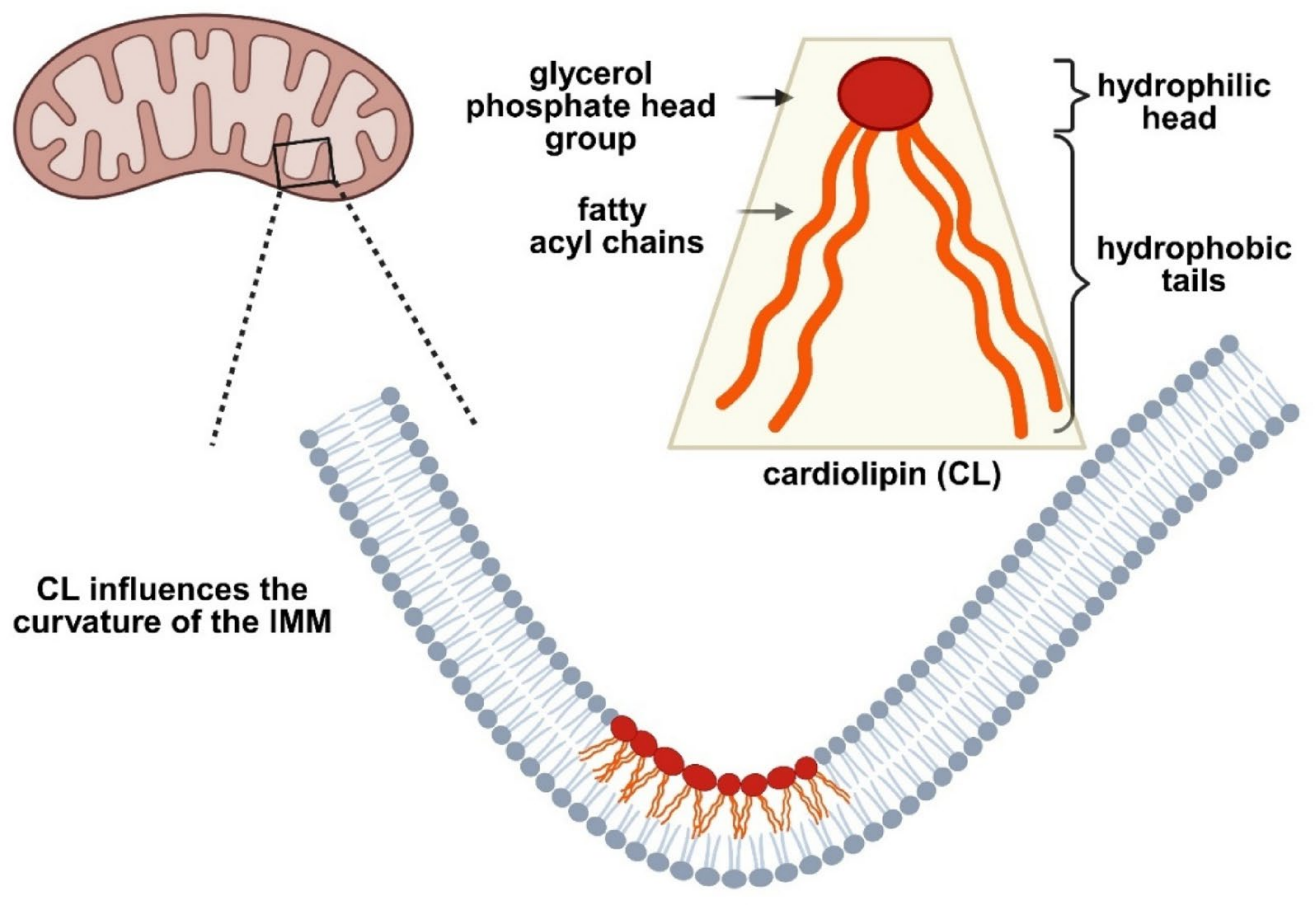
Schematic representation of CL conformation and localization. CL resides predominantly within the lipid bilayer of the inner mitochondrial membrane (IMM), where its unique conical shape contributes to membrane curvature and the stabilization of highly curved structures such as cristae. In addition, CL plays a crucial role in maintaining the structural organization of the IMM and supporting the optimal function of respiratory chain complexes. Created with BioRender.com

CL, a mitochondria-specific phospholipid, is central to this relationship. Localized predominantly to the IMM, CL accounts for ~ 20% of total mitochondrial lipids and is critical for crista folding, respiratory chain complex assembly, and overall mitochondrial structure [14, 35, 45, 69, 173, 231, 250]. Although normally restricted to the IMM, CL can externalize to the outer mitochondrial membrane (OMM) during stress [30, 173], where it functions as a signal for selective mitophagy [30, 69, 173]. In addition to its structural and bioenergetic roles, CL regulates fusion–fission dynamics, mitophagy, and mitochondria–lysosome communication. Dysregulation of these processes impairs respiration, enhances oxidative stress, and compromises neuronal survival (Fig. 3).

The unique conical geometry of CL—two phosphatidic acids linked to glycerol and four fatty acid chains—predisposes it to curved membrane regions within cristae [14, 69]. These physicochemical properties, including its high negative charge, confer strong affinity for the N-terminal amphipathic helix of α-syn [33, 48, 196]. Perturbations in CL concentration, structure, or oxidation status have been consistently associated with neurodegeneration, including PD [45, 57, 69, 173, 258].

Elevated CL levels promote α-syn insertion into membranes, altering fluidity and IMM dynamics [276] and enhancing the pore-forming activity of oligomers [77, 251]. This results in increased permeability, mitochondrial swelling, cytochrome c release, and impaired electron transport, partly due to reduced CL availability for respiratory chain complexes I, III, and IV [77, 110, 193, 201]. Importantly, α-syn enrichment at CL-rich sites not only destabilizes membranes but also accelerates their own oligomerization, further exacerbating mitochondrial dysfunction [45, 57, 69]. Experimental interventions confirmed this link: sequestration of CL with 10-N-nonyl acridine orange (NAO) protected isolated mitochondria from α-syn-induced swelling [77]. Consistently, α-syn species have been shown to impair complex I activity and increase reactive oxygen species (ROS) production in cellular and stem-cell models [29, 193, 201]. In addition to intrinsic mitochondrial defects, α-syn–CL interactions are increasingly recognized as triggers of neuroinflammatory cascades. The aggregation of α-syn, mitochondrial dysfunction, and microglial activation represent interconnected hallmarks of PD and related α-synucleinopathies. Recent studies have demonstrated that α-syn aggregates bind to CL on mitochondrial membranes, driving mitochondrial damage, mitophagy, and the release of damage-associated molecular patterns (DAMPs) that potently activate microglia [29, 151, 201]. Microglial responses are strongly aggregation dependent: fibrillar α-syn species induce robust proinflammatory signalling and further compromise mitochondrial integrity [107, 137, 168, 204].

**Fig. 4:**
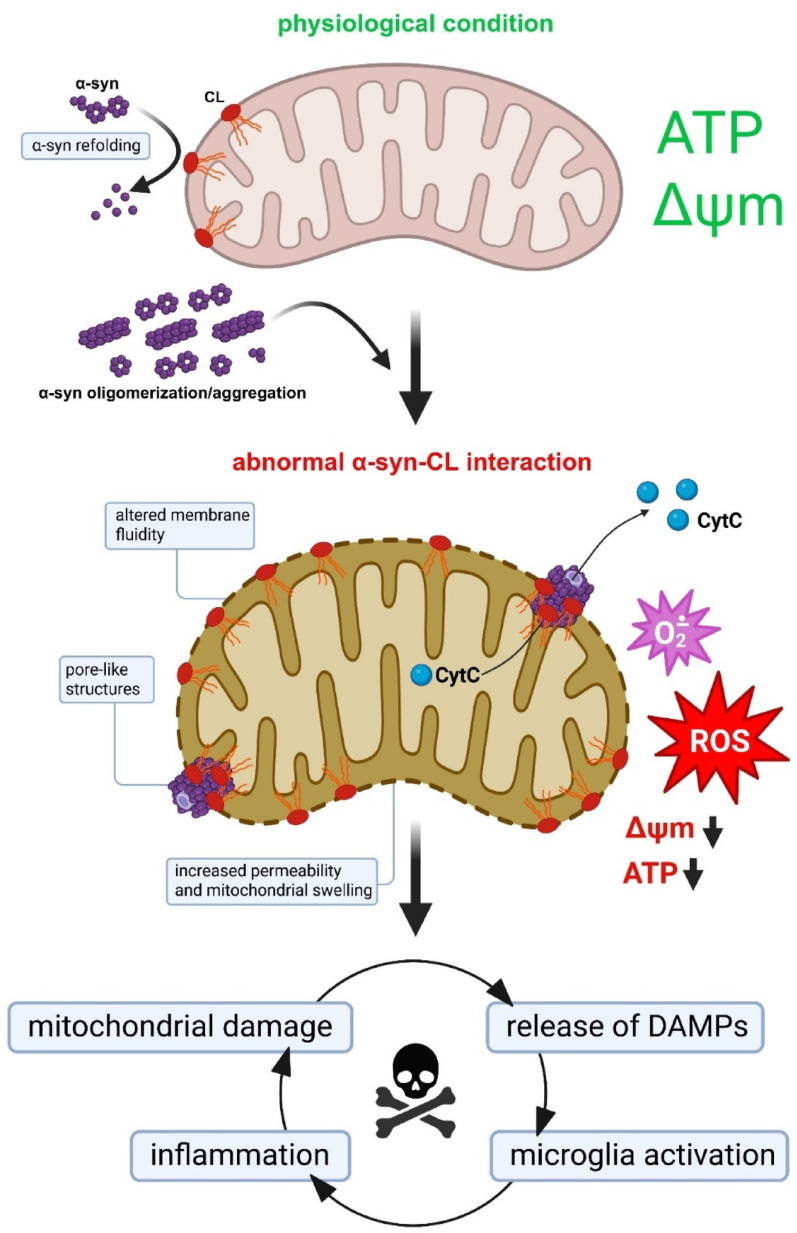
α-Syn-CL interaction drives mitochondria-dependent neurodegeneration. Aberrant α-syn binding to CL facilitates the formation of pore-like structures within the mitochondrial membrane, leading to membrane permeabilization, mitochondrial dysfunction, cytochrome c release, enhanced ROS generation, activation of pro-inflammatory signaling, and ultimately cell death. Created using BioRender.com

CL exposure and remodelling, therefore, act as critical nodes, simultaneously modulating α-syn aggregation and shaping microglial immune reactivity [272]. Evidence from human induced pluripotent stem cells (iPSCs)-derived neurons, transgenic mouse models, and proteomic analyses underscores the dual nature of CL: under physiological conditions, it may support α-syn refolding and mitochondrial quality control, whereas when dysregulated, it promotes aggregation, mitophagy, and cell death [95, 137, 204]. Microglia are not passive bystanders in this process. By amplifying inflammatory signalling and generating ROS, they reinforce mitochondrial stress, creating a vicious cycle in which α-syn aggregation, CL-driven dysfunction, and neuroinflammation perpetuate each other [107, 168, 185, 204, 225, 272]. Despite this strong evidence, unresolved questions remain, including the precise triggers for CL externalization and the bidirectional influences between mitochondrial dysfunction and inflammatory activation. Importantly, early studies suggest that therapeutic strategies aimed at increasing mitochondrial proteolysis or reprogramming microglial phenotypes could attenuate this cycle; however, their translation to the clinic remains in its infancy. Together, these findings support a model in which α-syn–CL interactions promote mitochondrial permeabilization, pore formation, and cell death pathways (Fig. 4).

In addition to membrane destabilization, emerging evidence points to CL as a regulator of α-syn condensation via LLPS. The intrinsically disordered nature of α-syn favours LLPS, and CL-rich microdomains may provide nucleation platforms that lower the threshold for condensate formation. These condensates, which are initially dynamic and reversible, under pathological conditions can undergo liquid-to-solid transitions, serving as intermediates toward toxic aggregates [53, 183, 191, 286]. This mechanism may help explain the emergence of conformational strains across α-synucleinopathies and their diverse clinical phenotypes [121, 128, 183, 294]. Membrane surfaces, including CL-enriched mitochondrial domains, act as active regulators of LLPS by concentrating proteins, providing spatial cues for nucleation, and modulating condensate stability [100, 222]. Molecular chaperones add an additional layer of control, yet their failure in disease may tip the balance toward pathological phase transitions.

Intriguingly, CL itself has been reported to undergo LLPS in cooperation with dynamin-related protein 1 (Drp1), a master regulator of mitochondrial fission [152, 186]. Drp1 recruitment to mitochondrial membranes is controlled by PTM modifications and interactions with OMM receptors (Mff, MiD49/51, Fis1) [91, 232]. Recent work has suggested that Drp1 and CL undergo phase separation to form droplets that regulate fission events [152, 186]. Disruption of this finely tuned process can result in abnormal mitochondrial fragmentation and may create a permissive environment for α-syn condensation. Thus, CL appears to intersect two pathogenic axes: impaired mitochondrial dynamics and α-syn aggregation.

Although direct mechanistic links between CL-driven LLPS and α-syn pathology remain to be fully elucidated, the convergence of evidence suggests that CL is more than a structural lipid. It functions as a molecular switch that determines whether α-syn remains in a physiological condensate state or progresses toward irreversible fibrillization. Understanding how the mitochondrial lipid composition influences this decision may be crucial in explaining selective neuronal vulnerability in PD.

Importantly, CL-α-syn interface is a potential therapeutic target. Strategies aimed at stabilizing CL metabolism, preserving membrane integrity, or preventing aberrant α-syn–CL interactions may offer novel avenues for disease modification. It also remains a matter of debate whether CL functions exclusively as a pathogenic trigger.

Moreover, alterations in CL composition and remodeling show promise as both diagnostic [57] and stratification biomarkers. CL profiling is already implemented clinically in the diagnosis of Barth syndrome (BTHS), where characteristic shifts in the monolysocardiolipin-to-cardiolipin (MLCL/CL) ratio provide a highly specific biochemical signature [126, 208]. Emerging evidence further indicates that disease-associated CL alterations may serve as biomarkers in FTD [181] and in experimental models of PD [253]. Consequently, comprehensive characterization of CL species and their remodeling dynamics in PD may not only yield mechanistic insights but also facilitate patient stratification based on mitochondrial lipid signatures, with potential implications for diagnosis, prognosis, and disease monitoring [57].

## CL-focused therapeutic interventions to mitigate α-syn–induced neurotoxicity

Reducing the toxicity of pathological α-syn remains a central therapeutic goal [52, 93, 171, 184, 221, 261, 266], and CL has emerged as a promising target [141, 173]. Interventions currently under investigation include stabilizing CL, preventing its oxidative damage, enhancing its biosynthesis and remodelling, and promoting targeted mitochondrial repair (Table 1). Although most remain at a preclinical stage, they illustrate the growing recognition of mitochondria—and particularly CL—as strategic nodes for disease modification in PD.

**Table 1:** CL-targeted interventions as potential disease-modifying strategies in PD

Therapeutic strategy	Mechanism of action	References
CL Stabilization: Elamipretide (ELAM) and SBT-272	A mitochondrial-targeting peptide that binds to CL and protects it from oxidative damage	[75, 182, 252]
Preventing CL Oxidation: MitoQ	A mitochondrion-targeted antioxidant that prevents CL from oxidative damage	[65, 238, 239]
Preventing CL Oxidation: SkQ1	A mitochondrion-targeted antioxidant that prevents CL from oxidative damage	[7, 195, 220]
Enhancing CL Biosynthesis: Tafazzin Gene Therapy and PUFA Supplementation	Enhance tafazzin function restoring CL homeostasis Improve and increase levels of CL restoring mitochondrial function	[92, 97, 104, 134, 229, 245, 252]

### CL stabilization

Among the most advanced approaches are CL-targeting peptides designed to restore mitochondrial bioenergetics. Elamipretide has been (also known as SS-31, MTP-131, or Bendavia) extensively studied in disorders associated with mitochondrial dysfunction [179, 192, 283, 293]. This Szeto-Schiller peptide penetrates the IMM, selectively binds CL, and stabilizes cristae architecture through electrostatic and hydrophobic interactions [242]. By doing so, it preserves respiratory chain activity, reduces cytochrome c peroxidase activity, suppresses ROS generation, and thereby limits the oxidative modifications that drive α-syn misfolding [162, 242, 252].

Recent preclinical work has indicated that elamipretide can directly modulate α-syn aggregation. For example, Stefaniak et al. (2024) reported the inhibition of α-syn fibrillization and the restoration of mitochondrial function in α-syn–challenged neuroblastoma cells [230]. These findings suggest that stabilizing CL not only supports mitochondrial bioenergetics but also interrupts the feedback loop between lipid oxidation and proteotoxic stress. Nevertheless, critical questions remain: does Elamipretide reduce CL externalization during α-syn stress, and do morphological changes in fibrils translate into sustained neuroprotection in vivo? Clinical data on PD are still lacking.

A related compound, SBT-272 (bevemipretide trihydrochloride), displays similar CL-binding properties but higher brain bioavailability and mitochondrial uptake, particularly in ALS models [75]. Whether these pharmacokinetic advantages translate into therapeutic benefits in PD remains to be determined.

### Preventing CL oxidation

Because CL is enriched in PUFAs, it is highly susceptible to peroxidation. Thus, mitochondrion-targeted antioxidants represent a rational therapeutic avenue [239]. MitoQ, a synthetic ubiquinone derivative, efficiently accumulates in mitochondria and remains in its reduced, antioxidant-active state [238]. In PD models, MitoQ protects against neuronal loss, striatal dopamine depletion, and mitochondrial dysfunction [78, 256]. Notably, it has been proposed to act as a CL-protective agent by preventing peroxidation of its FA chains [65].

SkQ1, a plastoquinone-based antioxidant, similarly localizes to the IMM and forms stable complexes with CL [8]. By preserving CL integrity, SkQ1 supports respiratory chain function and reduces mitochondrial and inflammatory stress in PD models [7, 175, 195, 220]. Together, these findings highlight mitochondrion-targeted antioxidants as potential modulators of both oxidative stress and α-syn aggregation, although their precise effects on CL metabolism require further clarification.

### Increased CL biosynthesis and remodelling

In addition to stabilization and protection, strategies to increase CL biosynthesis and remodelling may restore mitochondrial resilience in PD. CL is synthesized from phosphatidylglycerol and remodelled into its mature, functional form by tafazzin (TAZ) [159, 173, 194]. Loss of TAZ function, as observed in BTHS, results in defective CL remodelling and mitochondrial dysfunction [32, 104]. Insights from BTHS therapies—including Elamipretide and gene therapy, which restore Tafazzin activity—offer a conceptual framework for PD [189, 240, 241, 267].

Dietary and pharmacological supplementation with PUFAs represents another route to influence CL remodelling. N-3 fatty acids, such as alpha-linolenic acid (ALA), eicosapentaenoic acid (EPA), and docosahexaenoic acid (DHA), promote the incorporation of protective acyl chains into CL, stabilize mitochondrial membranes, and generate anti-inflammatory mediators [5, 18, 47, 92, 113, 131, 140, 146, 244]. In contrast, n-6–derived arachidonic acid (AA) yields proinflammatory eicosanoids, and both AA and DHA can interact directly with α-syn, influencing its aggregation dynamics [19, 20, 41, 62, 66, 105, 106, 116, 178, 216, 217]. These bidirectional interactions highlight the dual role of PUFA metabolism in modulating both α-syn aggregation and CL composition. Imbalances in PUFA intake or remodelling capacity may therefore exacerbate mitochondrial vulnerability.

Taken together, interventions that stabilize CL, prevent its oxidative damage, or restore its remodelling capacity represent promising mitochondria-directed strategies to counteract α-syn–induced toxicity. Although most approaches remain at the preclinical stage, they underscore the growing recognition of CL as a key therapeutic target in PD and highlight the need for rigorous validation in translational models to bridge the gap toward clinical application.

## Limitations in clinical translation of CL-directed mitochondrial interventions

Despite substantial progress in developing CL-focused and mitochondria-targeted therapies for PD and other α-synucleinopathies, translating these findings into clinical benefit remains challenging. Several biological and pharmacokinetic barriers continue to limit therapeutic efficacy. A major obstacle is the temporal discrepancy between preclinical and clinical intervention: while experimental studies typically administer treatments during early, reversible phases of mitochondrial dysfunction, patients entering clinical trials often present with advanced neurodegeneration and chronic mitochondrial impairment, substantially reducing the likelihood of meaningful therapeutic response [166].

Efficient drug delivery to mitochondria in the CNS is another major obstacle. The mitochondrion’s double-membrane architecture and strong negative membrane potential restrict access of therapeutic molecules; compounds that accumulate in mitochondria in vitro often fail to reach comparable concentrations in vivo [119, 143]. Classical targeting moieties such as triphenylphosphonium (TPP) conjugates suffer from nonspecific biodistribution, membrane-potential dependence and potential toxicity, while CL-binding constructs and peptide carriers achieve only partial delivery to the inner mitochondrial membrane. Even advanced carriers (e.g., CL-binding motifs or peptides such as SS-31) show limited in vivo success, frequently due to incomplete inner-membrane delivery or insufficient accumulation at the pathological site [99, 119, 190, 284, 289].

The blood–brain barrier (BBB) further constrains CNS exposure: many mitochondria-targeted compounds display poor BBB permeability, resulting in subtherapeutic brain concentrations. In vitro BBB models often oversimplify the in vivo environment and can overestimate CNS delivery. After systemic administration, rapid metabolism or peripheral sequestration may further reduce neuronal uptake and increase off-target effects [74, 167].

Translational obstacles are compounded by species- and tissue-specific differences in CL composition. Rodent and human tissues differ markedly in CL acyl-chain diversity, remodeling pathways and turnover rates; intra-tissue variability can exceed inter-species differences, complicating extrapolation from rodent lipidomics to human biology and potentially altering drug responses [169, 170, 226].

Finally, chronic manipulation of CL metabolism carries risks of unintended effects on mitochondrial structure and function, including altered cristae architecture, respiratory chain supercomplex organization, and mitophagy efficiency. These potential consequences highlight the need for careful assessment of long-term safety in translational studies.

Taken together, the limited clinical translation of CL-directed mitochondrial therapies stems from persistent obstacles in CNS penetration, intracellular and intramitochondrial targeting, pharmacokinetics, and species-dependent lipid biology. Resolving these challenges through innovative delivery technologies, precision targeting motifs, and model systems that accurately mirror human CL metabolism will be pivotal in determining whether cardiolipin-focused interventions can mature into effective complements to broader mechanistic strategies.

## Concluding remarks and future perspectives

CL has emerged as a pivotal determinant of PD pathology, acting at the intersection of mitochondrial biology, protein aggregation, and quality control pathways. Dysregulated interactions between CL and α-syn contribute to mitochondrial dysfunction, impaired mitophagy, and neuronal death. Accordingly, strategies aimed at stabilizing CL metabolism or preventing its oxidative remodelling hold considerable promise as mitochondrion-targeted therapies. However, despite encouraging preclinical evidence, clinical validation is still lacking, and it remains uncertain whether modulation of CL alone can produce durable neuroprotection in the complex landscape of PD.

Several conceptual challenges remain unresolved. First, the duality of CL as both a facilitator of mitophagy and a promoter of α-syn aggregation raises questions about context-specific effects. Under physiological conditions, CL externalization acts as a signal for selective mitochondrial clearance, whereas in stressed neurons, the same process may facilitate α-syn binding and aggregation. Whether these opposing outcomes are dictated by CL abundance, oxidation status, or cofactors such as chaperones remains unclear. A deeper understanding of this balance is critical for designing interventions that preserve the protective roles of CL without exacerbating pathogenic pathways.

Second, although LLPS has been increasingly implicated in the early steps of α-syn aggregation, the contribution of CL to condensate formation remains largely speculative. Evidence from other amyloidogenic proteins suggests that biomolecular condensates may both promote and inhibit aggregation, depending on local physicochemical conditions [124]. It is therefore plausible that CL-rich mitochondrial microdomains could act as nucleation platforms for α-syn condensates, but equally, they might serve as buffering sites that transiently sequester α-syn in a reversible state. Distinguishing between these possibilities represents a major gap in current knowledge. Methodologically, however, LLPS remains difficult to study. However, in vitro and biomimetic systems (e.g., CL-enriched liposomes or giant unilamellar systems) allow visualization of condensate formation and assessment of condensate dynamics via fluorescence recovery after photobleaching (FRAP) or related techniques, indicating that the operation of such condensates in neurons under physiological conditions is far more challenging. Moreover, differentiating reversible condensates from insoluble aggregates in living cells remains a major technical hurdle. From a therapeutic standpoint, direct manipulation of LLPS is still at an early conceptual stage. Because phase separation underlies essential physiological processes such as stress granule formation, global inhibition is likely to have deleterious effects. More realistic approaches may involve modulating the lipid environment (e.g., stabilizing CL and protecting it from oxidation), enhancing chaperone activity to preserve condensate fluidity, or regulating α-syn posttranslational modifications that influence phase behavior. At present, however, no interventions directly targeting LLPS in neurodegeneration are available, and this represents both a challenge and an opportunity for future research.

Third, it remains uncertain whether the modulation of CL metabolism in vivo can alter the disease trajectory. Most therapeutic strategies—such as CL-stabilizing peptides or mitochondrion-targeted antioxidants—have been evaluated in acute or reductionist models. Longitudinal studies in animal models that more faithfully reproduce progressive α-syn pathology will be essential to determine whether interventions targeting CL provide sustained benefits. Additionally, the extent to which systemic manipulation of lipid metabolism (e.g., through PUFA supplementation) translates into selective effects on neuronal CL pools requires clarification. Finally, the heterogeneity of PD itself poses a challenge. Distinct α-syn strains and clinical phenotypes may arise from differences in lipid environments, including CL composition and remodeling. If so, this suggests that CL signatures could serve not only as therapeutic targets but also as biomarkers to stratify patients. The development of sensitive imaging tools or lipidomic approaches to monitor CL status in vivo may therefore be a crucial step toward precision medicine in PD.

It also remains debated whether CL acts exclusively as a pathogenic trigger. On the one hand, CL-rich domains may buffer α-syn by transiently sequestering it into condensates, thereby delaying fibrillization; on the other hand, the same interaction may lower the threshold for pathological aggregation. This duality underscores the need for precise contextual studies to disentangle the protective and detrimental roles of CL.

In conclusion, CL occupies a central position at the crossroads of mitochondrial function, protein aggregation, and phase behavior. It acts not only as a structural lipid but also as a molecular switch that can bias α-syn either toward physiological interactions or pathological aggregation. Deciphering how this switch operates under different cellular contexts remains one of the most pressing questions in the field. Addressing these gaps—through mechanistic studies, biomarker development, and translational trials—offers the opportunity to transform our understanding of PD pathogenesis and to pioneer mitochondria-directed strategies that extend beyond symptomatic relief towards true disease modification. In the future, the integration of lipidomics, structural biology, and emerging imaging tools to visualize LLPS in living cells will be essential for dissecting these mechanisms at high resolution. Such approaches may ultimately pave the way toward precision mitochondrion-targeted therapies, where interventions are tailored not only to α-syn pathology but also to the lipid environment that shapes it.

## Data Availability

Data sharing is not applicable to this article, as no datasets were generated or analysed during the current study.

## Abbreviations

AA: Arachidonic acid
ALA: Alpha-linolenic acid
ALS: Amyotrophic lateral sclerosis
α-Syn: α-Synuclein
ATPase: Adenosine triphosphatase
BBB: Blood–brain barrier
BTHS: Barth syndrome
CL: Cardiolipin
CNS: Central nervous system
DAMPs: Damage-associated molecular patterns
DHA: Docosahexaenoic acid
DMPS: 2-Dimyristoyl-sn-glycero-3-phospho-L-serine
Drp1: Dynamin-related protein 1
EPA: Eicosapentaenoic acid
FRAP: Fluorescence recovery after photobleaching
FTD: Frontotemporal dementia
IMM: Inner mitochondrial membrane
iPSCs: Human induced pluripotent stem cells
LB: Lewy body
LLPS: Liquid–liquid phase separation
LN: Lewy neurites
LP: Lewy pathology
MLCL: Monolysocardiolipin
MSA: Multiple system atrophy
NAC: Nonamyloid-β component
NAO: 10-N-nonyl acridine orange
PD: Parkinson’s disease
PFF: Recombinant preformed fibril
POPA: 1-Palmitoyl-2-oleoyl-sn-glycero-3-phosphate
POPC: 1-Palmitoyl-2-oleoyl-sn-glycero-3-phosphocholine
PTMs: Posttranslational modifications
ROS: Reactive oxygen species
SARS-CoV-2: Severe acute respiratory syndrome coronavirus 2
SNpc: Substantia nigra pars compacta
TAZ: Tafazzin
